# The impact of preexisting comorbidities on receipt of cancer therapy among women with Stage I–III breast cancer in the Detroit Research on Cancer Survivors cohort

**DOI:** 10.1002/cam4.6456

**Published:** 2023-08-11

**Authors:** Sreejata Raychaudhuri, Jaclyn M. Kyko, Julie J. Ruterbusch, Stephanie S. Pandolfi, Jennifer L. Beebe‐Dimmer, Ann G. Schwartz, Michael S. Simon

**Affiliations:** ^1^ Department of Medical Oncology/Hematology University of Pittsburgh, Hillman Cancer Center Pittsburgh Pennsylvania USA; ^2^ Department of Oncology Karmanos Cancer Institute at Wayne State University Detroit Michigan USA; ^3^ Population Studies and Disparities Research Program Karmanos Cancer Institute Detroit Michigan USA

**Keywords:** advanced age, cancer treatment, comorbidities, racial disparities, Stage I–III breast cancer

## Abstract

**Purpose:**

Pre‐existing comorbidities play an important role in choice of cancer treatment. We retrospectively evaluated the relationship between pre‐existing comorbidities and receipt of local and systemic therapy in a cohort of Black women with Stage I–III breast cancer.

**Methods:**

The study population for analysis included 1169 women with Stage I–III disease enrolled in the Detroit Research on Cancer Survivors (ROCS) cohort. Information on comorbidities, socio‐demographic, and clinical variables were obtained from self‐reported questionnaires and the cancer registry. Comorbidities were analyzed individually, and comorbidity burden was categorized as low (0–1), moderate (2–3) or high (≥4). We used logistic regression analysis to evaluate factors associated with receipt of local treatment (surgery ± radiation; *N* = 1156), hormonal (*N* = 848), and chemotherapy (*N* = 680). Adjusted models included variables selected a priori that were significant predictors in univariate analysis.

**Results:**

Receipt of treatment was categorized into local (82.6%), hormonal (73.7%), and/or chemotherapy (79.9%). Prior history of arthritis and depression were both associated with a lower likelihood to receive local treatment, [odds ratio (OR), 95% confidence interval (CI), 0.66, 0.47–0.93, and 0.53, 0.36–0.78], respectively. Obesity was associated with higher likelihood of receiving hormonal therapy (OR: 1.64, 95% CI: 1.19, 2.26), and heart failure a lower likelihood (OR: 0.46, 95% CI: 0.23, 0.90). Older age (P_trend_ <0.01) and increasing co‐morbidity burden (P_trend_ = 0.02) were associated with lower likelihood of receiving chemotherapy.

**Conclusion:**

History of prior co‐morbidities has a potentially detrimental influence on receipt of recommended cancer‐directed treatment among women with Stage I–III breast cancer.

## 1 INTRODUCTION

Breast cancer is the most common cancer diagnosed among women in United States.^1^ In recent years, breast cancer incidence has been rising by 0.3% per year, however mortality rates have decreased by 1.3% per year.^1^, ^2^ There are known racial and ethnic disparities in outcomes for women with breast cancer, with Black women experiencing similar incidence but higher mortality compared to White women.^1^

Prior studies have shown that aging and history of multiple comorbidities negatively influences physician treatment recommendations for women with early‐stage breast cancer.^3^, ^4^, ^5^ Potential implications associated with altered treatment recommendations include lower and potentially less effective chemotherapy dosing in the setting of multiple comorbidities, concern about enhanced side effects, and the potential biological interplay between other diseases and cancer which may diminish the effectiveness of cancer directed treatment. The implications of altered treatment is especially true for elderly women who received delayed and non‐standard therapies, regardless of comorbidities, which negatively impacted outcomes.^3^, ^4^, ^5^

We evaluated the association between self‐reported, pre‐existing comorbidities and receipt of recommended local and systemic therapy in a cohort of Black women with Stage I–III breast cancer enrolled in the Detroit Research on Cancer Survivors (ROCS) study. With the extensive collection of data on sociodemographic, clinical and treatment information, the ROCS cohort provides a unique opportunity to study factors associated with receipt of treatment among Black cancer survivors.^6^ With increasing incidence and higher mortality from breast cancer in the Black community and a high comorbidity burden,^1^, ^2^, ^24^ a better understanding of factors that may influence choice and receipt of cancer‐directed treatment is crucial to understanding ongoing racial disparities in breast cancer outcomes. Other studies looked at mainly White women, such as that by Klepin et al.^13^ (87% White vs. 11% Black), Parise et al.^3^ with more than 50% White women, or Minicozzi et al.^4^ looking at cancer registries across nine European countries, which likely has few Black participants—hence this study provides valuable insight into an exclusively Black cohort. We expect these results to be generalizable to other urban Black populations in the United States.

## 2 METHODS

### 2.1 Study population

The population‐based ROCS study is the largest cohort study conducted exclusively among Black cancer survivors addressing multilevel determinants of poorer outcomes in this high‐risk population.^6^, ^7^ Briefly, Black men and women with invasive breast, lung, prostate, or colorectal cancer diagnosed since 2013, as well as endometrial cancer and other cancer sites diagnosed at less than age 50, since 2016, were identified through the Metropolitan Detroit Cancer Surveillance System (MDCSS). Survivors were sent letters inviting them to complete an enrollment survey by one of three methods; either online, by phone with a trained interviewer, or by written questionnaire.

As of November 20, 2020, 1408 breast cancer survivors were enrolled in the study. The current study is based on the first 1280 female breast cancer survivors who had complete data available for analysis. We excluded women diagnosed with American Joint Committee on Cancer (AJCC) 8th Edition stage 0, IV, or unknown stage (*N* = 105), and those who did not complete the medical history section of the questionnaire (*N* = 6), leaving 1169 women with AJCC Stage I–III breast cancer that were included in the final analysis. In regards to the methodology of survey completion, the survey was completed by 266 (22.8%) women online, 476 (40.7%) were interviewer‐assisted, and 427 (36.5%) were completed by written questionnaire. The median time from cancer diagnosis to study enrollment in the ROCS cohort was 21 months (Interquartile range: 13–35 months).

We evaluated three categories of cancer treatment identified through the MDCSS including localized treatment (surgery +/− radiation therapy), hormonal therapy, and chemotherapy. The study population for each category of treatment was based on the availability of treatment data and on NCCN recommendations for treatment. For localized treatment we included 1156 women after excluding 13 who had unknown information on receipt of radiation. For hormone therapy, we excluded 278 women for which hormone therapy was not recommended based on National Comprehensive Cancer Network (NCCN) guidelines^8^ as well as those with unknown hormone receptor status (HR) or missing information on receipt of hormone therapy (*N* = 43), resulting in 848 women included in this analysis. For chemotherapy, we excluded survivors with unknown HR/HER2 status and/or unknown lymph node status (*N* = 44), HR‐positive, human epidermal growth factor receptor 2 (HER2)‐negative breast cancer with unknown Onco*type* DX scores (*N* = 270), and survivors who were not recommended chemotherapy as per NCCN criteria (mentioned in detail below) (HR‐positive, HER2‐negative breast cancer; *N* = 175), leaving a sample of 680 women for the chemotherapy analysis. (Figure S1).

### 2.2 Study measures

Self‐reported characteristics at enrollment included gender, education, marital status, annual household income, health insurance coverage, smoking status, and participation in moderate or vigorous physical activity in the past 4 weeks. Participants were asked if a doctor ever told them that they had any of the following medical conditions including their corresponding age at onset: arthritis, emphysema/COPD, depression, diabetes, hepatitis, high cholesterol, hypertension, stroke, thyroid problem, heart attack, congestive heart failure (CHF), atrial fibrillation, or coronary artery disease. Participants were considered to have a pre‐existing comorbid condition at the time of cancer diagnosis if they answered in the affirmative to the specified condition, and the age at onset was less than the age at cancer diagnosis. In addition, if the age of onset was unknown, it was assumed that the diagnosis occurred prior to the cancer diagnosis as the patients were likely unable to recall a diagnosis made several years ago. Obesity was defined as a body mass index (BMI; body weight in kg/height in meters squared) of 30 or more calculated from self‐reported height and weight 1 year before cancer diagnosis. Our data set did not include any information on Eastern Cooperative Oncology Group (ECOG) performance status which is derived by clinicians in the office. Instead we hypothesized that self‐reported ability to perform moderate or vigorous physical activity (listed in Table 1) would potentially be associated with whether or not physicians were willing to recommend standard of care cancer treatment.

**TABLE 1:** Socio‐demographic and clinical characteristics of women with early stage breast cancer in the Detroit Research on Cancer Survivors cohort.

	*N*	%
Total	1169	100
Age at diagnosis		
<50	314	26.9%
50–64	521	44.6%
65+	334	28.6%
Age at diagnosis, mean (SD) (Range: 27–79)	57.1	(11.2)
Education		
Less than high school	90	7.8%
High school	254	22.0%
Some or 2‐year college degree	463	40.2%
4‐year college degree or above	345	29.9%
Marital status		
Married or living with a partner in a marriage‐like relationship	368	31.6%
Widowed	163	14.0%
Divorced or separated	313	26.9%
Never married	319	27.4%
Annual household income		
Less than $20,000	416	38.5%
$20,000–$59,999	433	40.1%
$60,000+	231	21.4%
Health insurance at the time of diagnosis		
No	14	1.2%
Yes	1122	98.8%
Physical activity defined as moderate or vigorous exercise at least once per week in the past 4 weeks		
No	537	46.2%
Yes	625	53.8%
Smoking status		
Never	656	56.5%
Former	354	30.5%
Current	151	13.0%
AJCC stage, 8th Edition		
I	604	51.7%
II	425	36.4%
III	140	12.0%
Diagnosis year		
2013–2014	356	30.5%
2015–2016	401	34.3%
2017–2018	340	29.1%
2019–2020	72	6.2%
Hormone receptor status		
ER+ or PR+, HER2+	136	11.6%
ER+ or PR+, HER2−	713	61.0%
ER−/PR−, HER2+	68	5.8%
Triple negative	210	18.0%
Unknown	42	3.6%
Oncotype DX score		
Low (recurrence score 0–17)	197	16.9%
Medium (recurrence score 18–30)	111	9.5%
High (recurrence score ≥ 31)	41	3.5%
Not reported or unknown	820	70.1%

*Note*: Values were not reported or were unknown for the following: education (*n* = 17), marital status (*n* = 6), income (*n* = 89), insurance (*n* = 33), physical activity (*n* = 7), smoking status (*n* = 8).

Abbreviation: SD, standard deviation.

Comorbidity burden was based on count of co‐morbid conditions, with 0–1 conditions categorized as “low,” 2–3 as “moderate,” and 4 or higher as “high.” The categorization of conditions as outlined was based on the available numbers in each category. Other cancer‐specific data were obtained from the MDCSS registry database and included age at diagnosis, diagnosis year, AJCC stage, HR/HER2 status, Onco*type* DX scores, and first‐course treatment: surgery (none, lumpectomy, mastectomy), radiation, hormone therapy, and chemotherapy. Receipt of radiation, hormonal therapy, or chemotherapy were classified as yes versus no.

Localized treatment, hormonal therapy, and chemotherapy were considered recommended if they met standard treatment guidelines including NCCN guidelines.^8^ Localized treatment with surgery +/− radiation was considered “recommended” for women who had lumpectomy and radiation for any stage of disease, mastectomy with or without radiation for T1–T2 stage and lymph node‐negative disease, and mastectomy with radiation for T3–T4 stage or lymph node‐positive disease. Hormone therapy was considered “recommended” for survivors with HR‐positive, HER2‐positive, or HR‐positive, HER2‐negative disease. Chemotherapy was considered recommended for survivors who had the following tumor characteristics: HR‐positive, HER2‐positive or HR‐negative, HER2‐positive or triple negative disease; HR‐positive, HER2‐negative, and lymph node‐positive disease, 50 years of age or younger at diagnosis with HR‐positive, HER2‐negative, lymph node‐negative disease and Onco*type* DX score of 26 or higher; and women older than 50 years at diagnosis with HR‐positive, HER2‐negative, lymph node‐negative disease and an Onco*type* DX score of 31 or higher.

### 2.3 Statistical analysis

All analyses were conducted using SAS software (version 9.4; SAS, Cary, NC) and an alpha value of 0.05 was set to determine statistical significance. The distribution of selected participant characteristics and cancer‐related factors were summarized using counts and percentages in Tables 1 and 2 and distributions by receipt of recommended treatments using counts and percentages in Tables 3, 4, 5. Logistic regression models were used to estimate odds ratios (OR) and 95% confidence intervals (CI) to quantify associations for age at diagnosis, comorbidity burden, pre‐existing individual comorbidities, and receipt of recommended treatment. Adjusted models included variables that were selected a priori and were significant predictors in univariate models using categories presented in Tables 1 and 2. The analysis for receipt of localized treatment was adjusted for marital status, annual household income, AJCC stage, and diagnosis year; the analysis for receipt of hormone therapy adjusted for survey method at enrollment and diagnosis year, and the analysis for receipt of chemotherapy was adjusted for AJCC stage, diagnosis year, and HR status. We assessed trends by including ordinal variables in the models for age and comorbidity.

**TABLE 2:** Cancer directed therapy received by women with early stage breast cancer in the Detroit Research on Cancer Survivors cohort.

	*N*	%
Total	1169	100
Treatments received		
Surgery		
No	38	3.3%
Yes	1131	96.7%
Radiation		
No	331	28.6%
Yes	825	71.4%
Hormone therapy		
No	510	43.7%
Yes	658	56.3%
Chemotherapy		
No	536	45.9%
Yes	633	54.1%
Local/regional treatment^a^		
No	201	17.4%
Yes	955	82.6%
Hormonal therapy^b^		
No	223	26.3%
Yes	625	73.7%
Chemotherapy^c^		
No	137	20.1%
Yes	543	79.9%

*Note*: Values were not reported or were unknown for the following: radiation (*n* = 13), hormone therapy (*n* = 1), local/regional treatment per recommendation (*n* = 13), hormone therapy per recommendation (*n* = 321), chemotherapy treatment per recommendation (*n* = 489). All treatment recommendations per NCCN guidelines and best clinical practice.

Abbreviations: AJCC, American Joint Committee on Cancer, 8th Edition; SD, standard deviation.

^a^ Recommended local/regional treatment (Surgery ± radiation) = lumpectomy + radiation for any stage of disease, mastectomy ± radiation for T1‐T2 stage and lymph node‐negative disease, and mastectomy + radiation for T3–T4 stage or lymph node‐positive disease.

^b^ Recommended hormonal therapy = HR‐positive, HER2‐positive, or HR‐positive, HER2‐negative disease.

^c^ Recommended chemotherapy = HR‐positive, HER2‐positive or HR‐negative, HER2‐positive or triple negative disease; HR‐positive, HER2‐negative, and lymph node‐positive disease, ≤50 years at diagnosis with HR‐positive, HER2‐negative, lymph node‐negative disease and Onco*type* DX score of 26 or higher; and women >50 years at diagnosis with HR‐positive, HER2‐negative, lymph node‐negative disease and an Onco*type* DX score of 31 or higher.

**TABLE 3:** The relationship between age at diagnosis and history of co‐morbid conditions and the receipt of recommended localized treatment in the Detroit Research on Cancer Survivors early stage breast cancer cohort.

	Receipt of recommended localized treatment^a^							
	Yes		No		Unadjusted		Adjusted^b^	
	*N*	Row %	*N*	Row %	OR	95% CI	OR	95% CI
Total	955	82.6%	201	17.4%				
Age at diagnosis								
<50	263	84.6%	48	15.4%	Ref		Ref	
50–64	435	84.6%	79	15.4%	1.01	(0.68, 1.48)	1.07	(0.70, 1.63)
65+	257	77.6%	74	22.4%	0.63	(0.42, 0.95)	0.72	(0.45, 1.15)
p‐trend						0.02		0.17
Comorbid conditions								
Comorbidity burden								
Low	269	83.8%	52	16.2%	Ref		Ref	
Moderate	379	84.8%	68	15.2%	1.08	(0.73, 1.60)	1.05	(0.68, 1.63)
High	307	79.1%	81	20.9%	0.73	(0.50, 1.08)	0.80	(0.52, 1.25)
p‐trend						0.09		0.29
Obesity								
No (BMI < 30)	383	81.3%	88	18.7%	Ref		Ref	
Yes (BMI≥30)	549	83.3%	110	16.7%	1.15	(0.84, 1.56)	1.22	(0.87, 1.72)
Arthritis								
No	560	85.6%	94	14.4%	Ref		Ref	
Yes	395	78.7%	107	21.3%	0.62	(0.46, 0.84)	0.66	(0.47, 0.93)
COPD or emphysema								
No	895	83.0%	183	17.0%	Ref		Ref	
Yes	60	76.9%	18	23.1%	0.68	(0.39, 1.18)	0.84	(0.45, 1.57)
Depression								
No	779	84.3%	145	15.7%	Ref		Ref	
Yes	176	75.9%	56	24.1%	0.59	(0.41, 0.83)	0.53	(0.36, 0.78)
Diabetes								
No	743	83.2%	150	16.8%	Ref		Ref	
Yes	212	80.6%	51	19.4%	0.84	(0.59, 1.19)	0.86	(0.58, 1.27)
Myocardial infarction								
No	909	82.7%	190	17.3%	Ref		Ref	
Yes	37	82.2%	8	17.8%	0.97	(0.44, 2.11)	1.14	(0.48, 2.73)
Congestive heart failure								
No	905	83.0%	185	17.0%	Ref		Ref	
Yes	41	75.9%	13	24.1%	0.65	(0.34, 1.23)	0.87	(0.42, 1.78)
Atrial fibrillation								
No	928	82.7%	194	17.3%	Ref		Ref	
Yes	18	81.8%	4	18.2%	0.94	(0.32, 2.81)	1.76	(0.48, 6.44)
Coronary artery disease								
No	929	82.7%	194	17.3%	Ref		Ref	
Yes	17	81.0%	4	19.0%	0.89	(0.30, 2.67)	1.23	(0.34, 4.51)
Hepatitis								
No	935	82.5%	198	17.5%	Ref		Ref	
Yes	20	87.0%	3	13.0%	1.41	(0.42, 4.79)	1.29	(0.35, 4.77)
High cholesterol								
No	621	82.4%	133	17.6%	Ref		Ref	
Yes	334	83.1%	68	16.9%	1.05	(0.76, 1.45)	1.09	(0.76, 1.56)
Hypertension								
No	387	83.6%	76	16.4%	Ref		Ref	
Yes	568	82.0%	125	18.0%	0.89	(0.65, 1.22)	1.00	(0.70, 1.42)
Stroke or cerebrovascular disease								
No	913	83.0%	187	17.0%	Ref		Ref	
Yes	42	75.0%	14	25.0%	0.61	(0.33, 1.15)	0.65	(0.32, 1.31)
Thyroid problem								
No	841	83.4%	167	16.6%	Ref		Ref	
Yes	114	77.0%	34	23.0%	0.67	(0.44, 1.01)	0.63	(0.39, 1.01)

*Note*: Values were not reported or were unknown for the following: obesity (*n* = 26), myocardial infarction (*n* = 12), congestive heart failure (*n* = 12), atrial fibrillation (*n* = 12), coronary artery disease (*n* = 12).

Abbreviations: CI, confidence interval; OR, odds radio.

^a^ Cases with known radiation status.

^b^ Adjusted models control for marital status, annual household income, AJCC stage, and diagnosis year.

**TABLE 4:** Associations between age at diagnosis and co‐morbid conditions and recommended hormone therapy for participants in the ROCS early stage breast cancer cohort.

	Receipt of recommended hormone therapy^a^							
	Yes		No		Unadjusted		Adjusted^b^	
	*N*	Row %	*N*	Row %	OR	95% CI	OR	95% CI
Total	625	73.7%	223	26.3%				
Age at diagnosis								
<50	149	71.0%	61	29.0%	Ref		Ref	
50–64	287	76.1%	90	23.9%	1.31	(0.89, 1.91)	1.50	(1.00, 2.24)
65+	189	72.4%	72	27.6%	1.08	(0.72, 1.61)	1.24	(0.81, 1.91)
p‐trend						0.80		0.42
Comorbid conditions								
Comorbidity burden								
Low	159	68.5%	73	31.5%	Ref		Ref	
Moderate	250	76.2%	78	23.8%	1.47	(1.01, 2.14)	1.47	(1.00, 2.16)
High	216	75.0%	72	25.0%	1.38	(0.94, 2.02)	1.42	(0.95, 2.12)
p‐trend						0.12		0.10
Obesity								
No (BMI < 30)	225	68.4%	104	31.6%	Ref		Ref	
Yes (BMI≥30)	387	77.4%	113	22.6%	1.58	(1.16, 2.17)	1.64	(1.19, 2.26)
Arthritis								
No	348	72.5%	132	27.5%	Ref		Ref	
Yes	277	75.3%	91	24.7%	1.16	(0.85, 1.58)	1.19	(0.86, 1.64)
COPD or emphysema								
No	582	74.2%	202	25.8%	Ref		Ref	
Yes	43	67.2%	21	32.8%	0.71	(0.41, 1.23)	0.78	(0.44, 1.37)
Depression								
No	517	74.3%	179	25.7%	Ref		Ref	
Yes	108	71.1%	44	28.9%	0.85	(0.58, 1.26)	0.82	(0.55, 1.22)
Diabetes								
No	474	72.6%	179	27.4%	Ref		Ref	
Yes	151	77.4%	44	22.6%	1.30	(0.89, 1.89)	1.29	(0.88, 1.91)
Myocardial infarction								
No	594	74.0%	209	26.0%	Ref		Ref	
Yes	25	71.4%	10	28.6%	0.88	(0.42, 1.86)	0.80	(0.37, 1.74)
Congestive heart failure								
No	597	74.7%	202	25.3%	Ref		Ref	
Yes	22	56.4%	17	43.6%	0.44	(0.23, 0.84)	0.46	(0.23, 0.90)
Atrial fibrillation								
No	606	73.9%	214	26.1%	Ref		Ref	
Yes	13	72.2%	5	27.8%	0.92	(0.32, 2.61)	1.26	(0.43, 3.63)
Coronary artery disease								
No	610	74.2%	212	25.8%	Ref		Ref	
Yes	9	56.3%	7	43.8%	0.45	(0.16, 1.21)	0.56	(0.20, 1.58)
Hepatitis								
No	609	73.4%	221	26.6%	Ref		Ref	
Yes	16	88.9%	2	11.1%	2.90	(0.66, 12.72)	3.00	(0.67, 13.37)
High cholesterol								
No	398	72.9%	148	27.1%	Ref		Ref	
Yes	227	75.2%	75	24.8%	1.13	(0.82, 1.55)	1.10	(0.79, 1.53)
Hypertension								
No	244	72.2%	94	27.8%	Ref		Ref	
Yes	381	74.7%	129	25.3%	1.14	(0.83, 1.55)	1.17	(0.85, 1.61)
Stroke or cerebrovascular disease								
No	593	73.5%	214	26.5%	Ref		Ref	
Yes	32	78.0%	9	22.0%	1.28	(0.60, 2.73)	1.31	(0.60, 2.86)
Thyroid problem								
No	548	74.0%	193	26.0%	Ref		Ref	
Yes	77	72.0%	30	28.0%	0.90	(0.58, 1.42)	0.90	(0.57, 1.43)

*Note*: Values were not reported or were unknown for the following: obesity (*n* = 19), myocardial infarction (*n* = 10), congestive heart failure (*n* = 10), atrial fibrillation (*n* = 10), coronary artery disease (*n* = 10).

Abbreviations: CI, confidence interval; OR, odds radio.

^a^ Cases who are recommended to receive hormone therapy with known hormone receptor and hormone therapy status.

^b^ Adjusted models control for survey method at enrollment and diagnosis year.

**TABLE 5:** Associations between age at diagnosis and co‐morbid conditions and recommended chemotherapy for participants in the ROCS early stage invasive breast cancer cohort.

	Receipt of recommended chemotherapy^a^							
	Yes		No		Unadjusted		Adjusted^b^	
	*N*	Row %	*N*	Row %	OR	95% CI	OR	95% CI
Total	543	79.9%	137	20.1%				
Age at diagnosis								
<50	204	86.8%	31	13.2%	Ref		Ref	
50–64	243	81.0%	57	19.0%	0.65	(0.40, 1.04)	0.62	(0.37, 1.03)
65+	96	66.2%	49	33.8%	0.30	(0.18, 0.50)	0.29	(0.17, 0.51)
p‐trend						<0.01		<0.01
Comorbid conditions								
Comorbidity burden								
Low	175	82.2%	38	17.8%	Ref		Ref	
Moderate	212	82.5%	45	17.5%	1.02	(0.64, 1.65)	0.89	(0.53, 1.49)
High	156	74.3%	54	25.7%	0.63	(0.39, 1.00)	0.55	(0.33, 0.91)
p‐trend						0.04		0.02
Obesity								
No (BMI < 30)	232	82.6%	49	17.4%	Ref		Ref	
Yes (BMI≥30)	296	77.9%	84	22.1%	0.74	(0.50, 1.10)	0.74	(0.48, 1.13)
Arthritis								
No	333	82.4%	71	17.6%	Ref		Ref	
Yes	210	76.1%	66	23.9%	0.68	(0.47, 0.99)	0.66	(0.44, 0.99)
COPD or emphysema								
No	511	80.1%	127	19.9%	Ref		Ref	
Yes	32	76.2%	10	23.8%	0.80	(0.38, 1.66)	0.96	(0.43, 2.14)
Depression								
No	423	79.2%	111	20.8%	Ref		Ref	
Yes	120	82.2%	26	17.8%	1.21	(0.76, 1.94)	1.02	(0.61, 1.69)
Diabetes								
No	442	80.8%	105	19.2%	Ref		Ref	
Yes	101	75.9%	32	24.1%	0.75	(0.48, 1.18)	0.72	(0.44, 1.18)
Myocardial infarction								
No	517	79.9%	130	20.1%	Ref		Ref	
Yes	20	76.9%	6	23.1%	0.84	(0.33, 2.13)	0.81	(0.30, 2.21)
Congestive heart failure								
No	515	80.0%	129	20.0%	Ref		Ref	
Yes	22	75.9%	7	24.1%	0.79	(0.33, 1.88)	0.62	(0.24, 1.57)
Atrial fibrillation								
No	528	80.1%	131	19.9%	Ref		Ref	
Yes	9	64.3%	5	35.7%	0.45	(0.15, 1.36)	0.57	(0.18, 1.86)
Coronary artery disease								
No	532	80.0%	133	20.0%	Ref		Ref	
Yes	5	62.5%	3	37.5%	0.42	(0.10, 1.77)	0.41	(0.09, 1.93)
Hepatitis								
No	536	79.9%	135	20.1%	Ref		Ref	
Yes	7	77.8%	2	22.2%	0.88	(0.18, 4.29)	0.97	(0.17, 5.55)
High cholesterol								
No	382	82.0%	84	18.0%	Ref		Ref	
Yes	161	75.2%	53	24.8%	0.67	(0.45, 0.99)	0.62	(0.41, 0.95)
Hypertension								
No	238	82.4%	51	17.6%	Ref		Ref	
Yes	305	78.0%	86	22.0%	0.76	(0.52, 1.12)	0.71	(0.47, 1.08)
Stroke or cerebrovascular disease								
No	519	80.1%	129	19.9%	Ref		Ref	
Yes	24	75.0%	8	25.0%	0.75	(0.33, 1.70)	0.83	(0.34, 2.01)
Thyroid problem								
No	482	80.2%	119	19.8%	Ref		Ref	
Yes	61	77.2%	18	22.8%	0.84	(0.48, 1.47)	0.64	(0.35, 1.17)

*Note*: Values were not reported or were unknown for the following: obesity (*n* = 19), myocardial infarction (*n* = 7), congestive heart failure (*n* = 7), atrial fibrillation (*n* = 7), coronary artery disease (*n* = 7).

Abbreviations: CI, confidence interval; OR, odds radio.

^a^ Cases who are recommended chemotherapy with known hormone receptor status, lymph node status, and Oncotype DX score.

^b^ Adjusted models control for AJCC stage, diagnosis year, and hormone receptor status.

## 3 RESULTS

Table 1 shows the demographic and clinical characteristics of women in the study cohort that was derived from participant self‐report. The mean age at diagnosis was 57 years, Standard deviation (SD) 11.2 and interquartile range 27–79. About 70% of participants reported at least some college education, 1/3 were married, 78.6% had an average household income of less than $60,000 and 98% had medical insurance. Regarding lifestyle factors, 43.5% were either current or former smokers and 53.8% were able to perform at least moderate to vigorous exercise once a week. Stage at diagnosis as listed in MDCSS showed 51.7% had AJCC stage I, 36.4% stage II, and 12% stage III disease. Other tumor characteristics that was derived from the MDCSS included 18% with triple negative disease and 3.5% with a high Oncotype DX recurrence score of ≥31.

Table 2 shows the distribution of first‐line local treatment received (derived from the MDCSS) showing that 96% of the women had breast surgery and 71% had radiation therapy. The proportion of women overall who received cancer‐directed treatment included 82.6% who received localized treatment, 73.7% hormonal treatment, and 79.9% chemotherapy.

Table 3 shows the un‐adjusted and adjusted models evaluating the association between age at diagnosis and self‐reported history of prior co‐morbidities and receipt of recommended localized treatment. In the multivariable adjusted models, there was no significant relationship between age (P_trend_ = 0.17) or comorbidity burden (P_trend_ = 0.29) and receipt of localized treatment, however history of arthritis and history of depression were both associated with a lower chance of receiving localized treatment (OR: 0.66, 95% CI: 0.47, 0.93 and OR: 0.53, 95% CI: 0.36, 0.78), respectively; there was no significant relationship between any of the other co‐morbid conditions and receipt of localized therapy.

Table 4 shows the model for receipt of recommended hormone therapy, and Table 5 for receipt of chemotherapy. After multivariable analyses, history of obesity was associated with a greater likelihood for receipt of hormonal therapy (OR: 1.64, 95% CI: 1.19, 2.26) and history of CHF was associated with a lower likelihood of receipt of hormonal therapy (OR: 0.46, 95% CI: 0.23, 0.90). For chemotherapy, after multivariable analysis, increasing age and increasing comorbidity burden were both associated with a lower likelihood of receipt of chemotherapy, (P_trend_ <0.01) and (P_trend_ = 0.02), respectively. In addition, women with a history of arthritis (OR: 0.66, 95% CI: 0.44, 0.99) and a history of hypercholesterolemia (OR: 0.62, 95% CI: 0.41, 0.95) were also less likely to receive chemotherapy. None of the other self‐reported co‐morbid conditions were associated with receipt of hormonal therapy or chemotherapy.

## 4 DISCUSSION

We evaluated the relationship between pre‐existing comorbidities and receipt of recommended cancer‐directed treatment in a cohort of Black survivors with early‐stage invasive breast cancer in an attempt to better understand factors associated with receipt of less than adequate cancer‐directed therapy. As expected, the majority of women in the ROCS cohort received recommended standard treatments, however there was a mixed relationship regarding receipt of different therapy modalities based on prior medical history. In ROCS, women with a history of either depression or arthritis were less likely to received standard localized therapy, women with obesity were more likely to receive hormonal therapy, and women with CHF were less likely to receive hormonal therapy. Negative predictors for receipt of chemotherapy included increasing age and increasing number of co‐morbid conditions, as well as a personal history of arthritis or hypercholesterolemia.

While several studies have looked at the relationship between prior co‐morbidity and breast cancer survival and mortality,^18^, ^19^, ^20^, ^21^, ^22^, ^23^ few have looked specifically at the relationship between increasing age, or history of specific medical conditions and receipt of treatment.^3^, ^4^, ^5^ Consistent with other studies, we found that both advanced age and history of co‐morbid medical conditions has a negative impact on subsequent receipt of chemotherapy and that selected co‐morbidities influence receipt of hormonal therapy or localized treatment.^3^, ^4^, ^5^ In one report of 20,177 women with triple negative breast cancer from the California Cancer registry (2000–2015),^3^ women with high co‐morbidity burden defined by a Charlson comorbidity index (CCI) of 2 or greater versus CCI‐0^9^ had higher breast‐cancer specific mortality. In addition, women with high CCI with any stage at diagnosis, were less likely to receive chemotherapy, had reduced use of lumpectomy + radiation (Stages 2–4) and reduced rate of mastectomy for Stage 3 disease.^3^ Also noted in the California Cancer Registry study, the proportion of Black women with CCI 2+ was almost twice as high as those with CCI of 0 (20.4% vs. 10.7%) as opposed to a similar proportion of White women with CCI 0 or CCI of 2 (51.2% vs. 57.6%).^3^

In the ROCS cohort, as data needed to construct the CCI variable was not collected, we used a count of prior co‐morbid conditions as a proxy for CCI. Despite the lack of data on CCI, our results are consistent with those reported by other studies which used CCI.^3^, ^4^ In a cohort of elderly breast cancer survivors, Parise et al.,^3^ demonstrated lower receipt of chemotherapy among older patients and those with a higher co‐morbidity burden. Garg et al. also reported that older age and increased co‐morbidity burden was associated with an increased likelihood for treatment stoppage.^10^ Others also reported less use of axillary lymph node dissection and radiation therapy in older women.^11^ In a similar analysis from nine European countries, older women were more likely to receive delayed and non‐standard treatments for their breast cancer and less likely to be offered breast conservation surgery and radiation.^4^ Similar findings showing negative impact of age and increasing comorbidity burden on receipt of standard cancer treatments, and consequently on outcomes, were replicated in many other analyses^14^, ^15^, ^16^, ^17^, ^18^, ^19^, ^20^, ^21^, ^22^, ^23^ including in a meta‐analysis of 60 studies.^5^

Potential explanations for the reported lower use of cancer‐directed therapy among individuals who had more co‐morbid conditions include the potential for physician concern or perception that older age and greater co‐morbidity burden is more likely to result in potentially dangerous and/or life‐threatening side effects from chemotherapy.^12^ It is also possible that patients who have multiple co‐morbid medical conditions have a higher likelihood to refuse treatment. Our data provides no specific explanations for why any particular treatment such as history of arthritis or history of hypercholesteremia might influence the recommendation or completion of chemotherapy, although again history of any co‐morbid medical condition might raise both physician and patient related concerns for added toxicity on top of the usual side effects associated with cancer treatment and thereby influence treatment decision making.^13^ Choice of cancer treatment is a complicated process taking into account numerous clinical factors, physician perception of a patient's ability to tolerate treatment and other aspects including perceived patient compliance and willingness to take treatment. The consistency of our findings with those of other studies however, suggests that the presence of co‐morbidities among women with early stage breast cancer might influence recommendations for cancer‐directed treatment, and emphasizes the importance of collaboration with primary care to optimize treatment of other medical conditions among women with breast cancer. Better treatment and control of co‐morbid medical conditions could potentially positively impact the optimal care of women with early stage breast cancer.

The strengths of our analysis include the large well‐established cohort of Black cancer survivors and the extensive data collection on a group which has been under‐represented in other studies.^3^, ^4^, ^5^, ^10^, ^11^, ^13^, ^15^, ^16^, ^17^, ^18^, ^19^, ^20^, ^21^, ^22^, ^23^ An analysis in a Black survivorship cohort is an important addition to the literature as other studies only included 10%–20% of Black women.^3^, ^13^ The importance of ROCS is also exemplified by the fact that Black cancer survivors have been shown to have worse outcomes after cancer diagnosis than other groups.^24^ It is also important to study the relationship between prior co‐morbid medical conditions and treatment in a Black survivorship cohort given that Blacks generally have higher rates of co‐morbid conditions than other groups.^24^ Lastly, the ROCS data set provides a comprehensive listing of socio‐demographic and clinical information derived through linkage with the MDCSS cancer registry database.

Limitations of our analysis include the retrospective data collection and possibility for recall bias particularly in regard to reports of co‐morbid medical conditions. In addition, we did not have information on performance status from a patient survey or a cancer registry which is commonly used by physicians to assess whether or not patients should get systemic chemotherapy. We felt however that the methods used to collect clinical information in ROCS can be justified in that it roughly approximates data collected from patients in the medical office, and we also used information on cancer diagnoses and treatment available in a population based cancer registry. There may however be misclassification and possibly under‐reporting of hormonal and chemotherapy in the MDCSS as that treatment is largely administered in the outpatient setting and not completely captured in the registry. Despite the fact that we did not collect all of the information necessary to construct a CCI, our results mimic those of other studies where CCI was used as a measure of the impact of co‐morbid medical conditions,^3^ justifying our analytic decisions. As indicated above, choice of cancer‐directed treatment is a complicated process involving medical providers, patients and care‐givers and a direct cause—and effect relationship between any of the studied variables including medical history and rationale for treatment recommendation or whether treatment was completed cannot be made. Other limitations include a possible survivor bias in that healthier cancer survivors are more likely to participate in ROCS, although the focus of our analysis on women with early stage breast cancer lessens this possibility.

In summary, in the ROCS cohort, age and medical history were associated with receipt of various forms of cancer‐directed therapy. These findings suggest that collaboration with primary care providers to optimize the medical care of co‐morbidities in women with early breast cancer could favorably impact the completion of cancer‐directed treatment.

## AUTHOR CONTRIBUTIONS

**Sreejata Raychaudhuri:** Conceptualization (equal); data curation (equal); formal analysis (equal); investigation (equal); methodology (equal); resources (equal); supervision (equal); validation (equal); writing – original draft (equal); writing – review and editing (equal). **Jaclyn M Kyko:** Data curation (equal); formal analysis (equal); writing – review and editing (equal). **Julie J. Ruterbusch:** Data curation (equal); formal analysis (equal); funding acquisition (equal); investigation (equal); methodology (equal); writing – review and editing (equal). **Stephanie S. Pandolfi:** Data curation (equal); formal analysis (equal); writing – review and editing (equal). **Jennifer Beebe‐Dimmer:** Funding acquisition (equal); writing – review and editing (equal). **Ann Schwartz:** Funding acquisition (equal); writing – review and editing (equal). **Michael Simon:** Conceptualization (equal); formal analysis (equal); investigation (equal); methodology (equal); project administration (equal); resources (equal); supervision (equal); validation (equal); writing – original draft (equal); writing – review and editing (equal).

## FUNDING INFORMATION

This work was supported by the National Cancer Institute of the National Institutes of Health (U01 CA199240 and CA199240‐04S1), the Epidemiology Research Core and National Institutes of Health Center Grant P30CA022453 awarded to the Karmanos Cancer Institute at Wayne State University, and the Metropolitan Detroit Cancer Surveillance System.

## CONFLICT OF INTEREST STATEMENT

The authors have no relevant financial or non‐financial interests to disclose.

## ETHICS STATEMENT

This study was reviewed and approved by Wayne State University's Institutional Review Board (#050417M1F).

## INFORMED CONSENT

Informed consent was obtained from all participants included in the study.

## Supporting information


Figure S1.

## Data Availability

The datasets generated during and/or analyzed during the current study are not publicly available due to patient confidentiality reasons but are available from the corresponding author on reasonable request.
