# Supplementary material for: The impact of preexisting comorbidities on receipt of cancer therapy among women with Stage I–III breast cancer in the Detroit Research on Cancer Survivors cohort
Source: Cancer Med. 2023 Aug 11;12(18):19021–32. doi: 10.1002/cam4.6456 (PMC10557862; doi:10.1002/cam4.6456)
Supplement: Supplementary file 1 — Figure S1. [file CAM4-12-19021-s001.docx]

**Supplemental Figure 1**. Flow diagram summarizing the research design

**Excluded cases with stage 0, IV and unknown stage invasive breast cancer**

N=105

**Comorbid condition(s) information available**

N=1,169

**Early stage (I-III) invasive breast cancer**

N=1,175

**Eligible and enrolled breast cancer patients identified in MDCSS with data available**

N=1,280

**Excluded cases who skipped the comorbidity section of the ROCS questionnaire**

N=6

Tables 1-2

Table 5

Table 4

Table 3

**Excluded cases with unknown radiation status**

N=13

**Localized treatment analysis sample**

N=1,156

**Excluded cases who are not recommended to receive hormone therapy or have unknown hormone receptor or hormone therapy status**

N=321

(ER-/PR-, HER2+) (N=68)

Triple negative (N=210)

Unknown hormone receptor status (N=42)

Unknown hormone therapy status (N=1)

**Hormone therapy analysis sample**

N=848

**Excluded cases with unknown data**

N=44

Unknown hormone receptor status (N=41)

Unknown lymph node status (N=2)

Unknown hormone receptor and lymph node status (N=1)

**Known hormone receptor and lymph node status**

N=1,125

**Excluded cases who are not recommended chemotherapy or have unknown Oncotype DX score**

N=445

(ER+ or PR+, HER2-) and unknown oncotype DX score (N=270)

(ER+ or PR+, HER2-), N0, ≤50 years at diagnosis, and oncotype DX score <26 (N=33)

(ER+ or PR+, HER2-), N0, 51+ years at diagnosis, and oncotype DX score <31 (N=142)

**Chemotherapy analysis sample**

N=680

Abbreviations: MDCSS – Metropolitan Detroit Cancer Surveillance System; ROCS – Research On Cancer Survivors
